# Low Arousing Positive Affect Broadens Visual Attention and Alters the Thought-Action Repertoire While Broadened Visual Attention Does Not

**DOI:** 10.3389/fpsyg.2016.01652

**Published:** 2016-10-25

**Authors:** Daniel T. Jäger, Jascha Rüsseler

**Affiliations:** ^1^Department of Psychology, University of BambergBamberg, Germany; ^2^Bamberg Graduate School of Affective and Cognitive Science, University of BambergBamberg, Germany

**Keywords:** visual attention, thought-action repertoire, Broaden-and-Build Theory, emotion, cognition

## Abstract

The Broaden-and-Build Theory states that positive emotions broaden cognition and therefore build personal resources. However, missing theoretical precision regarding the interaction of the cognitive processes involved offers a variety of possible explanations for the mechanisms of broadening and building. In Experiment 1 we tested the causality assumption which states that positive emotions first broaden visual attention which in turn leads to broadened cognition. We examined the effects of a broadened, narrowed or neutral attentional scope of 72 subjects (30 men) on their momentary thought-action repertoire. Results showed that there were no significant differences between groups regarding the breadth or the content of the thought-action repertoire. In Experiment 2 we studied the non-causality hypothesis which assumes a non-causal relationship between cognitive processes. We did so by investigating the effects of negative, neutral, and positive affect on the visual attentional scope of 85 subjects (41 men) in Experiment 2a, as well as on the thought-action repertoire of 85 participants (42 men) in Experiment 2b. Results revealed an attentional broadening effect in Experiment 2a but no differences between groups concerning the breadth of the thought-action repertoire in Experiment 2b. However, a theory driven content analysis showed that positive affect promoted social actions. Thus, our results favor the non-causality assumption. Moreover, results indicate that positive emotions do not target personal resources in general but rather resources associated with social behavior. In conclusion, we argue that the Broaden-and-Build Theory should be refined.

## Introduction

Evolution has not only shaped the human’s body (e.g., Shubin et al., 1991, 1997), but also the cognitive system (e.g., Aboitiz, 1992; Toupo et al., 2015). Emotions are a good example of how evolution shaped the cognitive system. They are believed to be “the neurocomputational adaptions that have evolved in response to the adaptive problem of matching arrays of mechanism activation to the specific adaptive demands imposed by alternative situations” (Tooby and Cosmides, 2008, p. 117). Thus, every emotional episode seems to serve a specific function by eliciting predetermined action tendencies. The fear of being stalked, for instance, prepares the body and the mind to deal quickly and efficiently with a potentially life-threatening situation. It does so by mobilizing all kinds of different programs such as individual goals, motivational priorities, information-gathering strategies, perceptual mechanisms, memory, attention, physiological activation, communication, emotional expressions, and behavioral tendencies (for a review see Tooby and Cosmides, 2008). However, Fredrickson and Cohn (2008) point out that the functional adaption model has a bias toward negative emotions. Action-tendencies elicited by positive emotions tend to be unspecific. Thus, the adaptive benefit of positive emotions is currently unclear.

Fredrickson’s (2001) Broaden-and-Build theory provides a theoretical framework of the adaptive functions positive emotions may serve. According to the model, positive emotions broaden cognition and, in turn, lead to broadened momentary thought-action repertoires. This results, when accumulating, in the building of intellectual, physical, and social resources. In other words, as their vision and cognition widen, people see more possible actions around them, like engaging in meaningful social conversations, building social bonds etc. Social resources in turn increase the likelihood of coping more effectively with adverse situations in the future making it more likely to get help from other people. Thus, positive emotions unfold their adaptive benefit over the long run (for a detailed review see Fredrickson, 2001). Furthermore, the Broaden-and-Build Theory predicts that the building of personal resources increases the frequency of positive emotional experiences and, therefore, triggers an upward spiral toward emotional well-being (Fredrickson and Joiner, 2002). Negative emotions have a rather oppositional effect on cognition and result in narrowed thought-action repertoires or specific action-tendencies.

**FIGURE 1:**
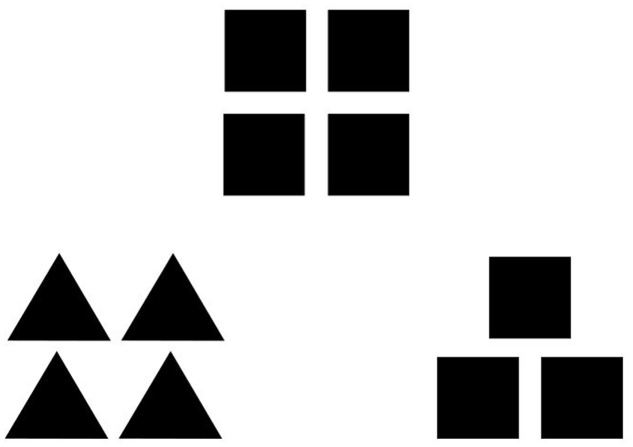
**Example for a global–local target item used in both experiments and by Fredrickson and Branigan (2005).** Participants had to indicate which of the two comparison figures below they found more similar to the standard figure above.

In order to put the Broaden-and-Build Theory to empirical test, Fredrickson and Branigan (2005) conducted two seminal experiments. In both experiments short film clips were used to induce either amusement, contentment, a neutral feeling, anger, or anxiety. Experiment 1 focused on the effects of positive emotions on the scope of visual attention, measured by a Global–Local-Visual Processing Task adapted from Kimchi and Palmer (1982). Participants were asked to decide which of the two figures below was more similar to the figure above (see **Figure 1**). If participants chose the geometrical figure with the same global configuration, their attentional focus was assumed to be broad. If they chose the figure with the same local geometrical form, their attentional focus was assumed to be narrow. In Experiment 2 the thought-action repertoire was measured by a Twenty Statement Test (TST). Participants were asked to write down all the actions they would like to do at that moment after having watched the respective film clip. The TST had exactly twenty blanks that could have been filled out. Results indicated that participants experiencing a positive emotion chose the global geometrical configuration more often in Experiment 1 and wrote down more actions in Experiment 2 compared to participants in the neutral condition. Moreover, participants experiencing a negative emotion wrote down less actions in Experiment 2 than participants in the neutral or positive condition. Taken together, results supported the theoretical framework of the Broaden-and-Build Theory.

However, there is still missing theoretical precision regarding how these various cognitive effects relate to one another (Lyubomirsky, 2000). Fredrickson offers two possible explanations for the cognitive broadening effect. However, she deliberately has not contrasted or named them, yet. We will call these two explanations the non-causality and the causality assumption. The non-causality assumption presumes that positive emotions guide a variety of cognitive processes at the same time, however, causality among these processes is not established. In Fredrickson’s and Branigan’s (2005, p. 315) own words this consideration “states that positive emotions broaden the scopes of attention, cognition, and action, widening the array of percepts, thoughts, and actions presently in mind.” The causality assumption instead establishes causality by postulating broadened attention as a mediator variable and therefore the essential variable for the cognitive broadening effect (Conway et al., 2013, p. 18), i.e., positive emotions would no longer be the crucial variable for broadening cognition and eventually building resources, but rather a broadened attentional focus. Consequently, positive emotions would be one out of many variables alongside, for example, low motivational intensity, as supposed by Harmon-Jones et al. (2013) that could broaden attention and foster the building of personal resources in the long run.

When looking at empirical evidence for the explanation that positive emotions alter a lot of different cognitive processes at the same time without one process being causally responsible for another one, there are contradicting findings regarding the broadening effect of positive emotions. On the one hand some studies induced positive emotions and observed broadening effects, among others, on memory by increasing memory for the central and peripheral details (Yegiyan and Yonelinas, 2011), on visual attention shown by longer peripheral fixation time in an eye tracking experiment (Wadlinger and Isaacowitz, 2006), or on cognitive flexibility indicated by lower switch costs between tasks (Lin et al., 2014). Furthermore, building effects of positive emotions with regard to three different personal resources could be observed performing a path analysis of a questionnaire study conducted among army wives (Dolphin et al., 2015). Thus, when looking at these studies, positive emotions indeed seem to have a broadening and building effect. On the other hand, there are studies that cannot find a cognitive broadening and building effect of positive emotions and therefore contradict the previously mentioned studies. Positive emotions did not influence spatial attention in a spatial pre-cueing paradigm (Tipples and Sharma, 2000) or the scope of attention using a Flanker Task (Finucane and Whiteman, 2007), for example. An explanation for this can be found in recent research which indicates that it might not be the valence of an emotion that leads to a broadening or narrowing of the attentional focus, but its motivational intensity (Gable and Harmon-Jones, 2010, 2012; Harmon-Jones et al., 2012, 2013). Thus, when approach motivation is manipulated independently of emotional valence, results show that high motivational intensity accounts for a narrowing of the attentional scope. A look at the basic experiments by Fredrickson and Branigan (2005) reveals that only positive emotions low (e.g., amusement, contentment) and only negative emotions high in motivational intensity (e.g., anger, anxiety) were induced. In contrast to that, pictures of delicious desserts, for example, induced positive emotions high in approach motivation while at the same time narrowing the scope of attention (Gable and Harmon-Jones, 2008). Moreover, another body of research also supports the Broaden-and-Build Theory. However, at the same time it indicates that arousal and task characteristics of the Global–Local-Visual Processing Task might be variables that could explain the previously mentioned contradicting findings. Under the umbrella of the affect as information approach Gasper and Clore (2002) found that participants in happier moods were more likely to use an accessible global concept than participants in a sad mood when reproducing a drawing from memory. Furthermore, in a second experiment they found that participants in sad moods were more likely to attend to local information in a Global–Local-Visual Processing Task. Hence, they concluded that positive affect privileges global, category-level attention more than negative affect does which goes along with predictions of the Broaden-and-Build Theory. On the contrary, Fredrickson and Branigan (2005) assume that global–local tasks measure the momentary scope of attention and not attention to global or local features. This assumption is especially debatable when looking at the origins of the Global–Local-Visual Processing Task grounded in research on the primacy of holistic processing and Gestaltism but not on the attentional breadth (Kimchi, 1992). Thus, it is questionable whether the Global–Local-Visual Processing Task really measures the momentary attentional scope but rather the extend of attention to global or local features of a stimulus. Furthermore, other variables like arousal (Vogt et al., 2008) or the personal relevance of the presented material (Grol et al., 2014) should be taken into account when interpreting results regarding attention. In attentional control, for example, arousal and affect interact with one another such that low arousing negative affect produces the best performance in an attentional blink task whereas high arousing negative affect produces the weakest performance. However, for positive affect arousal does not impact the performance at all (Jefferies et al., 2008). This interaction effect can also be observed in research on memory and emotion. Greene et al. (2010) found a memory enhancement effect in positive mood high-arousal and in negative mood low-arousal states relative to the other emotional states. Hence, the interaction of valence and arousal seems to be important when trying to understand the emotional enhancement of memory. Kensinger and Corkin (2004) even argue that valence and arousal in regard to emotional memory are processed by distinct neural processes such that arousal is dependent on an amygdala-hippocampal network whereas valence is supported by a prefrontal cortex-hippocampal network. Taken together, these findings do not necessarily contradict the Broaden-and-Build Theory as a whole, but rather extend it and take motivational intensity and arousal into account as well. Gable and Harmon-Jones (2010, p. 11) delimit arousal and motivational intensity from one another such that “motivational intensity, or the impetus to act, is related directly to arousal, but unlike arousal, motivation always has action implications (even if they are vague).” Hence, empirical findings show that the Broaden-and-Build Theory’s predictions are too general and, therefore, should be refined in terms of which specific state affects which specific cognitive function.

Furthermore, there is a lack of empirical evidence regarding the explanation assuming causality between attention and the thought-action repertoire. However, in creativity research, a relation between trait broad attention and trait creativity has been postulated (Kasof, 1997; Lin et al., 2013), indicating potential causality. In line with this theorizing, Fredrickson (2001) describes that being creative is one part of a broadened thought-action repertoire. As mentioned earlier, trait breadth of attention and creative performance seem to relate to one another (Kasof, 1997; Lin et al., 2013). Memmert (2007) found in a real world experimental attentional broadening intervention that instructions that broadened attention improved creative performance in ball sport games. However, there are also studies where a broader scope of attention is not linked to enhanced creative performance (e.g., Healey and Rucklidge, 2005). Nevertheless, the methodologically most convincing experiments by Friedman et al. (2003) established a causal relationship between broadened visual attention and enhanced creative performance in a divergent thinking task. This leads to the conclusion that external visual attentional broadening primes internal conceptual attentional broadening. Friedman et al. (2003) observed that the induction of a broad attentional focus enhances creativity. However, some methodological shortcomings like missing adequate control conditions or a missing check of the attentional manipulation hamper interpretation of the results. To sum up, an empirical basis of the link between positive emotions, attention, and the thought-action repertoire, is still missing. However, creativity research supports the assumption of broadened visual attention improving creative performance since some studies found increased creativity after broadening attention (Kasof, 1997; Friedman et al., 2003; Memmert, 2007). Although this research provides some promising findings, experiments are lacking proper control conditions.

Two experiments were conducted to address the raised issues. In order to make sure that the same concepts as in the Broaden-and-Build Theory were addressed, we designed both experiments using a similar methodology as the experiments reported by Fredrickson and Branigan (2005). Experiment 1 aimed at putting the causality assumption that positive emotions broaden attention which in turn broadens the thought-action repertoire to empirical test. In other words, this experiment tested the direct effects of the attentional scope on the thought-action repertoire. Although this experiment was conducted within the framework of the Broaden-and-Build Theory of positive emotions, emotions were not part of the experiment since we intended to investigate causal relations among cognitive processes known to be influenced by emotions. In short, the main idea was to induce a broad, narrow, or neutral attentional state and observe its effects on the thought-action repertoire. Experiment 2 addressed the previously described contradicting empirical findings regarding the cognitive broadening effect of positive emotions. We tried to replicate Fredrickson and Branigan’s (2005) results using a similar methodology, and in addition performed a theory driven content analysis of the Twenty Statements Test. In doing so, we tested the previously mentioned non-causality assumption among cognitive processes.

## Method and Results

### Experiment 1

#### Overview

**FIGURE 2:**
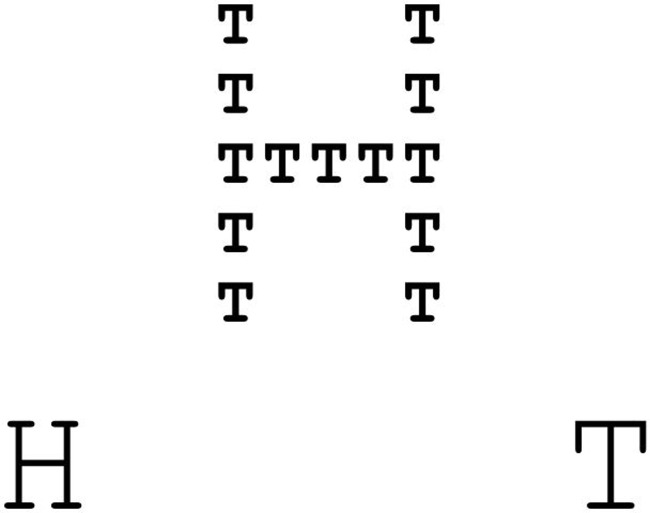
**Item example of the Navon Induction Task.** Participants either had to respond to the big letter or to the small letters depending on experimental condition.

Experiment 1 used a three group between subject design to test whether the scope of visual attention influences the cognitive thought-action repertoire. To this end, we employed a Navon Induction Task adapted from Perfect et al. (2008). For this task, Navon Letters were used (big letters composed of many small letters; for an example see **Figure 2**). Participants either had to indicate the identity of the big letter which should induce a broad attentional scope, or they had to indicate the identity of the small letters which should induce a narrow attentional scope. Subjects in the control condition did not complete the induction task. In order to ensure that the attention induction worked out as intended (manipulation check), participants took a Global–Local Visual Processing Task adapted from (Dale and Arnell, 2013, p. 397) which is similar to the tasks used by Kimchi and Palmer (1982) or Fredrickson and Branigan (2005). As described previously, participants had to decide which of the comparison figures, matching either on the global or local level, was more similar to the standard figure above (for an item example see **Figure 1**). The number of global and local choices indicated the momentary breadth of the attentional scope. In other words, the more often participants chose the figure matching on the global level the broader the momentary attentional scope was. Finally, the thought-action repertoire was gaged using the TST (Fredrickson and Branigan, 2005). Participants were asked to complete the sentence “I would like to …” with up to twenty things they would have liked to do at this moment. In order to better understand the broadening effect of action urges we performed a theory driven content analysis of the written material.

In short, we designed Experiment 1 to test effects of a broadened or narrowed visual attentional scope on the thought-action repertoire. We expected the broadening condition to display a globally biased attentional scope and the narrowing condition to display a locally biased attentional scope each compared to the neutral condition. In line with previous research on creativity (Friedman et al., 2003; Memmert, 2007) participants in the broadening condition should show a broadened overall thought-action repertoire score as well as writing down more social and more resource building actions compared to the neutral condition (for a detailed description see the Method section). In contrast, we expected participants in the narrowing condition to show a narrowed overall thought-action repertoire score as well as writing down less social and less resource building actions compared to the neutral condition.

#### Participants

Data was collected from 79 adults of which five were excluded due to a depression or anxiety disorder, one because of an acute migraine attack while testing, and another one due to misunderstanding the experimental procedure. Thus, the relevant sample consisted of 72 adults (30 men) aged between 17 and 44 (*M*_age_ = 23.3 years, *SD* = 4.3 years) who had not studied psychology or worked in the field of psychology. Participants were randomly assigned to experimental conditions (broadening, narrowing, neutral) so that each group comprised 24 participants. Subjects were recruited via e-mail and had normal or corrected to normal vision. Furthermore, five were ambidextrous, seven were left-handed, and 70 participants had at least an educational degree of a German high school diploma. Among all participants 20 vouchers each worth 10€ were raffled off 1 month after the study ended.

Both studies (Experiments 1 and 2) were conducted in accordance with the Declaration of Helsinki. Participants gave their written informed consent and were told that they could refrain from the study at any point without consequences. The study protocol was approved by the ethic commission of the University of Bamberg.

#### Material

##### Navon Induction Task

In order to induce a broad or narrow scope of attention participants completed a computerized version of the Navon Induction Task adapted from Perfect et al. (2008). Participants had to respond to 30 Navon Letters generated by one letter on the local level that was used to form another letter on the global level (see **Figure 2**). As local letters F, H, L, P, S, T, or E were used to build E, F, H, L, or T on the global level. Local and global letters did never match. The black inked Navon letters were placed in the middle of a 304-pixel × 228-pixel white rectangle with the two comprising black inked letters in the bottom right and left corner (see **Figure 2**). The Navon Letters had a height of 2.9 cm and a width of 2.0 cm whereas the corner letters had a height of 0.8 cm and a width of 0.6 cm. These stimuli were presented in the center of a 27-inch computer screen with a dark gray background. Participants were instructed to respond to the global letters in the broadening condition and to the local letters in the narrowing condition. Letter to response key matching was indicated by two small letters in the right or left corner of the display (see **Figure 2**). Moreover, participants kept a constant viewing distance of approximately 70 cm to the computer screen. In total, 30 Navon letters were presented in a randomized order. The stimuli remained on the screen until a response was executed. During the Inter-Stimulus-Interval of 1.0s a small fixation dot was presented. Correct responses were counterbalanced for the right and left key. Furthermore, pretesting revealed that time pressure leads to a narrowing of the momentary attentional scope. Thus, participants in the broadening condition were told that response time did not matter, whereas participants in the narrowing condition were instructed to respond as fast and accurate as possible. Participants in the control condition did not complete the task at all. Stimulus presentation for all computerized parts of Experiments 1 and 2 was controlled using Presentation Version 17.0 software^1^. Responses were collected with a Cedrus RB 830 response box^2^.

##### Global–Local Visual Processing Task

In order to check whether attention manipulation worked out as intended an adapted computerized version of the Global–Local Visual Processing Task (Kimchi and Palmer, 1982; Fredrickson and Branigan, 2005; Dale and Arnell, 2013) was administered to participants. Over the trials, eight test triads and 16 filler triads were presented in randomized order. The figures in each test triad comprised 3–4 small (8 mm × 8 mm) square or triangle figures on the local level that formed a bigger (19 mm × 19 mm) square or triangle on the global level. For the previously described target stimuli, both of the comparison figures in the bottom left and right corner matched the standard figure in the upper middle. One of the comparison figures matched the standard on the global level (the overall shape), and the other one matched on the local level (the smaller shape; see **Figure 1** for an example). The figures in each filler triad were composed of 3–4 small (8 mm × 8 mm) circles, squares, triangles, or crosses (local level) that formed a bigger (19 mm × 19 mm) square or triangle (global level). In the filler triads, just one comparison figure matched the standard figure either on the global or on the local level. The other comparison triad did not match the standard figure on a local or global level. Triads were presented on a 304 pixel × 228 pixel white rectangular background presented in the middle of a 27 inch computer screen with a dark gray background. Participants were instructed to choose the comparison figure that seemed more similar to the standard figure. They indicated their response by pressing the right or the left key on a two-key button box. Half of the 16 filler triads matched the top figure on the global level whereas the other half matched the top figure on the local level. Key presses in response to filler items were counterbalanced for the right and left key. The more often a participant chose a comparison figure matching the global configuration of the standard figure, the broader the attentional scope was assumed to be and vice versa for the local choices. Since only target items comprised comparison figures matching on the local and global level, only responses to the eight target items in each block were used to assess the momentary scope of attention, i.e., the momentary scope of attention was measured by counting the number of global choices to the target items resulting in a global score ranging from 0 to 8.

##### Twenty Statement Test (TST)

In order to measure the momentary thought-action repertoire an adapted version of the TST was employed. Participants were instructed to write down all the things they would like to do at the present moment. The paper in front of them comprised 20 blank lines each beginning with “I would like to …”. The more ideas a participant wrote down the broader the momentary thought-action repertoire was assumed to be. Thus, the momentary thought-action repertoire was measured by counting the numbers of filled in blank lines resulting in an overall score ranging from 0 to 20. Moreover, we performed a two dimensional content analysis using two independent raters unaware of the purpose of the experiment. Since Fredrickson (2001) underlines the importance of social actions within other resource building actions, we instructed both raters to categorize the named action-urges along the sociality dimension. In the second dimension, the resource dimension, we chose to look at resource building actions, being part of the proposed upward spiral of positive emotions, as well as resource protecting actions being part of the proposed downward spiral of negative emotions (Fredrickson, 2001). The sociality dimension consisted of two subcategories best described as social actions and non-social actions. Social actions were defined as actions that particularly mention or need by definition at least another person, like “having dinner with friends” or “talking on the phone.” Non-social actions were defined as actions that did not mention another person being involved, like “having dinner” or “watching TV.” The resource dimension comprised three subcategories best described as resource building actions, resource protecting actions, and indifferent actions. Resource building actions were defined as actions that are potentially improving the body’s fitness, intellectual performance, social bonding, or psychological resilience. Resource protecting actions were defined as actions that potentially protect physical, social, psychological, or intellectual resources, like “running away,” “getting help.” The indifferent subcategory was defined as actions that could not clearly be classified as belonging to one or the other resource subcategory, like “smoking a cigarette” or “stop time.” Interrater reliability calculated by using Pearson correlations was at least above 0.90 for all subscales.

#### Procedure

Participants were welcomed and seated inside a noise-canceling cabin in front of a 27 inch computer screen with a two-keyed button box in front of them. The cabin that lowered the sound level (-40 dB) was necessary because Kasof (1997) showed that the scope of attention and creative performance can be confounded by surrounding noises. Participants were randomly assigned to the conditions (broadening, narrowing, and neutral). First, they completed a Demographic Questionnaire. During a training phase participants in the broadening and narrowing condition were first familiarized with the Navon Induction Task and afterward with the Global–Local Visual Processing Task. Participants in the neutral condition were just familiarized with the Global–Local Visual Processing Task. Furthermore, participants were told to use the button box with both index fingers. In the broadening and narrowing condition participants completed the experiment in the following order: Demographic Questionnaire, Training phase, Navon Induction Task, Global–Local Visual Processing Task, TST, Global–Local Visual Processing Task. The neutral condition had almost the same experimental order except that there was no Navon Induction Task. We decided to employ the Global–Local Visual Processing Task twice for two reasons. First, we conducted the task after the completion of the TST because we wanted to make sure that participants remained in the induced state while completing the TST. Second, we measured the induced state before the TST because we also wanted to show that the visual attentional broadening effect existent after completing the TST was not due to the completion of the TST. The only way to show this, is by conducting two measurements that do not differ significantly from one another. After testing, participants were debriefed and were given the opportunity to sign up for the lottery of 20 vouchers each worth 10€.

#### Results

Since we conducted four separate ANOVAs in Experiment 1, we employed the Bonferroni correction in order to reduce the risk of false positive results. Thus, the adjusted critical *p*-value for each ANOVA was *p* = 0.0125 in order to maintain an experiment wise alpha level of α = 0.05.

Mean numbers of global choices in the Global–Local-Visual Processing Task were administered to a two-way ANOVA for mixed groups. There was a significant main effect for the factor GROUP, *F*(2,69) = 5.91, *p* = 0.004, η_p_ = 0.146. *Post hoc* Tukey Tests revealed that the broadening condition had significantly more global choices than the neutral condition, *p* = 0.027, as well as significantly more global choices than the narrowing condition, *p* = 0.005. The neutral and the narrowing condition did not differ significantly from one another, *p* = 0.828 (for means see **Table 1**). Furthermore, there was no significant main effect for the factor TIME, *F*(2,69) = 3.57, *p* = 0.063, η_p_ = 0.049 and no interaction effect of TIME and GROUP, *F*(2,69) = 1.34, *p* = 0.27, η_p_ = 0.037.

**Table 1:** Mean numbers of global choices in the Global–Local Visual Processing Task for experimental groups and Time of Measurement in Experiment 1.

	Broadening (*n* = 24)	Narrowing (*n* = 24)	Neutral (*n* = 24)
Time 1	5.04 ± 2.85	2.25 ± 2.55	2.67 ± 2.68
Time 2	4.33 ± 3.16	2.08 ± 2.55	2.58 ± 2.95


*Data are presented as means ± SD.*

The mean number of ideas participants wrote down in the TST were submitted to a one-way ANOVA. There were no significant differences between the broadening, *M* = 7.08, *SD* = 3.95, the narrowing, *M* = 6.29, *SD* = 5.03, and the neutral condition, *M* = 5.33, *SD* = 6.24, *F*(2,69) < 1. The mean numbers of ideas participants wrote down coded as social as well as the mean numbers coded as resource building were analyzed with a one-way ANOVA. Regarding social actions there were no significant differences between the broadening, *M* = 1.13, *SD* = 1.70, the narrowing, *M* = 0.77, *SD* = 1.27, and the neutral condition, *M* = 0.38, *SD* = 0.752, *F*(2,69) = 2.06, *p* = 0.135. Concerning resource building actions there were no significant differences between the broadening, *M* = 6.13, *SD* = 3.62, the narrowing, *M* = 5.68, *SD* = 4.97, and the neutral condition, *M* = 4.96, *SD* = 4.31, *F*(2,69) < 1.

#### Discussion

The purpose of Experiment 1 was to examine effects of visual attentional broadening and narrowing on the thought-action repertoire. This was accomplished by inducing a broad or narrow visual attentional focus and measuring effects on the thought-action repertoire afterward. Furthermore, the momentary attentional scope was gaged after the attention induction and after the thought-action repertoire measurement to ensure stability of the induced attentional scope. Results revealed that induction of the attentional scope worked for the broadening condition, but not for the narrowing condition. Therefore, results can only be interpreted for the effects of a broader attentional focus on the momentary thought-action repertoire. Furthermore, the attentional scope remained stable throughout the course of the experiment. Hence, results show that broadening the visual scope of attention has no effects on the content and the breadth of the momentary thought-action repertoire.

### Experiment 2

#### Overview

Experiment 2 was designed to examine the non-causality assumption among cognitive processes by replicating and extending Fredrickson’s and Branigan’s (2005) studies. Thus, we collected data in one session administering two experiments as Fredrickson and Branigan (2005) did, however, we used other film clips and performed a theory driven content analysis of the TST afterward. Experiment 2a used a three group between subject design in order to examine effects of negative and positive emotions on the visual attentional scope. Therefore, participants watched a short film clip that aimed at inducing negative, neutral or positive affect. Immediately afterward participants completed the Global–Local Visual Processing Task that we used in Experiment 1 (see **Figure 1**). Thereafter, participants filled in the Emotion Report Form originally used by Fredrickson and Branigan (2005) in order to examine if emotion induction worked as intended. Experiment 2b used a three group between subject design in order to examine effects of negative and positive emotions on the thought-action repertoire. Thus, participants watched a short film clip again, however, not the one they had watched before. Immediately afterward participants completed the TST used in Experiment 1 to measure the momentary thought-action repertoire They were instructed to write down all actions they would like to do given the emotion induced in the previous film. Subsequently, the Emotion Report Form used in Experiment 2a was administered again to check whether emotion manipulation worked out. Participants performed both Experiments (2a and 2b) in a randomized order.

We expected the positive film group to show a globally biased attentional scope and the negative film group to show a locally biased attentional scope each compared to the neutral condition. Experiment 2b examined the effects of negative and positive affect on the momentary thought-action repertoire. In line with Fredrickson and Branigan (2005) we expected the positive film group to write down more overall action urges compared to the neutral film group and the negative film group to write down less overall action urges compared to the neutral film group. Additionally, regarding the content analysis, we expected the positive film group to write down more social actions and more potentially resource building actions compared to the neutral film group. In contrast, the negative film group was expected to name less social actions and more resource protecting actions than the neutral film group.

#### Participants

Data was collected from 96 adults that did not take part in the first experiment. Data of three participants had to be excluded due to a depression disorder in both experiments. In Experiment 2a three participants were excluded from analysis due to data collection errors, and another five participants had to be excluded since emotion induction did not work for them as intended. Thus, the remaining sample in Experiment 2a consisted of 85 adults (41 men) aged between 18 and 31 (*M*_age_ = 21.9 years, *SD* = 2.9 years). Participants were randomly assigned to experimental conditions so that the negative film group comprised 28 participants, the neutral film group 30, and the positive film group 27. In Experiment 2b eight participants were excluded because emotion induction did not work. Thus, the relevant sample in Experiment 2b consisted of 85 adults (42 men) aged between 18 and 31 (*M*_age_ = 22.1 years, *SD* = 3.0 years). Participants were randomly assigned to experimental conditions so that the negative film group comprised 28 participants, the neutral film group 28, and the positive film group 29. For both experiments all subjects were recruited via e-mail and had normal or corrected to normal vision. Furthermore, eleven were left-handed and all participants had at least an educational degree of a German high school diploma. All participants either received course credit or 5€.

#### Material

##### Emotional film clips

In order to induce negative, neutral, or positive emotions participants watched a short film clip. For inducing negative affect, we showed a film clip called “lights out” (2 min 33 s) in which a woman wants to go to bed but keeps seeing shadows when switching off the lights. She gets more and more afraid reaching the film clip’s climax at the very end finishing with a disgusting shock moment. In the neutral experimental condition, we used a film clip called “Presentation” (3 min 11 s) in which a man summarizes the newest features of a presentation software. Lastly, for inducing positive affect we utilized a film clip called “laughing baby” (2 min) in which a toddler keeps laughing at his father who is ripping a sheet of paper into pieces.

In order to gain further insight into the films’ characteristics, 21 adults not taking part in the main experiment rated the extend of positive feelings, negative feelings, and arousal experienced during each individual film clip immediately after watching it. Ratings on each of the three dimensions ranged from “not at all” to “very much” on a 7-point Likert scale.

Mean scores of negative affect, positive affect, and arousal ratings were each subjected to a one-way repeated measures ANOVA with the factor FILM. Analyzing positive affect ratings resulted in/showed a significant main effect, *F*(2,40) = 47.8, *p* < 0.001, η_p_ = 0.71. *Post hoc* pairwise comparisons revealed that the positive film group, *M* = 5.43, *SD* = 1.36, showed significantly more positive affect than the neutral film group, *M* = 2.43, *SD* = 1.47, *p* < 0.001, as well as significantly more positive affect than the negative film group, *M* = 2.14, *SD* = 1.28, *p* < 0.001. Looking at the negative affect ratings there was a significant main effect, *F*(2,40) = 56.7, *p* < 0.001, η_p_ = 0.74. *Post hoc* pairwise comparisons revealed that the negative film group, *M* = 5.19, *SD* = 1.44, showed significantly more negative affect than the neutral film group, *M* = 2.57, *SD* = 1.43, *p* < 0.001, as well as significantly more negative affect than the positive film group, *M* = 1.52, *SD* = 1.12, *p* < 0.001. The neutral and the positive film group also differed significantly from one another, *p* = 0.002. Arousal ratings also displayed a significant main effect, *F*(2,40) = 43.3, *p* < 0.001, η_p_ = 0.68. *Post hoc* pairwise comparisons revealed that the negative film group, *M* = 4.76, *SD* = 1.84, reported significantly more arousal than the neutral film group, *M* = 1.48, *SD* = 0.81, *p* < 0.001 and the positive film group, *M* = 2.19, *SD* = 1.29, *p* < 0.001. However, the neutral and the positive film group did not differ significantly from one another, *p* = 0.11.

Taken together, these findings imply that main effects of valence cannot be interpreted for negative affect but for positive affect only as the neutral film differed significantly from the negative film with regard to arousal ratings but not from the positive film.

##### Emotion Report Form

Subjective experiences of certain emotions were assessed using a German translation of the Emotion Report Form adapted from Fredrickson and Branigan (2005). Participants were asked to rate each emotion (amusement, anger, anxiety, contentment, disgust, fear, happiness, sadness, and serenity) on a 9-point Likert scale (0 = none, 8 = a great deal) with regard to the extent of feeling that particular emotion while watching the film clip. Furthermore, ratings for amusement, contentment, happiness, and serenity were combined resulting in the overall positive affect scale, as well as anger, anxiety, disgust, fear, and sadness resulting in the overall negative affect scale.

In Experiment 2b, the momentary thought-action repertoire was assessed using the previously described Twenty-Statement-Test. However, we changed the instructions and used a German translation of the instructions Fredrickson and Branigan (2005) gave their participants. First, after viewing the film but before completing the Twenty-Statement-Test we asked participants to describe in one or two words the strongest emotion felt while watching the film. Afterward we told participants to step away from the details of the film and

“*… take a moment to imagine being in a situation yourself in which this particular emotion would arise (the one you wrote on the previous page). Concentrate on all the emotion you would feel and live it as vividly and as deeply as possible. Given this feeling, please list all the things you would like to do right now*.” (Fredrickson and Branigan, 2005, p. 320).

Scoring was conducted as described in the methods section of Experiment l.

#### Procedure

Participants were welcomed, filled out a demographic questionnaire and provided their informed consent at the beginning of the testing session. Then they were seated in a well-lit room in front of a 27-inch computer screen and were asked to remain in a stable viewing distance of approximately 70 cm. Participants were instructed only to use their two index fingers for responding with the two keyed button box in front of them. In addition, participants put on noise-canceling headphones. They were asked to keep the headphones on until the testing session ended in order to control for distracting noises. Afterward participants completed Experiments 2a and 2b. While these fully computerized testing sessions the experimenter left the lab in order to counteract social desirable responses as well as possible distraction effects produced by the experimenter. In Experiment 2a there were three possible film clips. Thereafter, in Experiment 2b there were only two possible film clips since we did not want participants to watch the same film clip twice. When also counterbalancing the order of Experiments 2a and 2b this results in 3 × 2 × 2 = 12 possible orders. In Experiment 2a, participants were familiarized with the Global–Local-Visual Processing Task practicing on two local and two global fillers which were presented in a randomized order. Second, participants watched the assigned film clip and completed the Global–Local-Visual Processing Task immediately afterward. Then participants were asked to fill in the Emotion Report Form. After a short break participants continued with Experiment 2b or vice versa. In Experiment 2b, participants watched the assigned film clip and completed the Twenty-Statement-Test immediately afterward. As a final step participants were instructed to fill in the Emotion Report Form. After testing, participants were debriefed and either received course credit or 5€ for participation.

#### Results

##### Experiment 2a

Since we conducted two separate ANOVAS in Experiment 2a, we employed the Bonferroni correction in order to reduce the risk of false positive results. Thus, the adjusted critical p-value for each ANOVA was *p* = 0.025 in order to maintain an experiment wise alpha level of α = 0.05.

###### Manipulation check

In order to check whether emotion induction worked as intended in Experiment 2a mean scores of the negative affect scale as well as mean scores of the positive affect scale were both submitted to a one-way ANOVA with the factor FILM. The Levene’s test indicated that the assumption of homogeneity of variance was violated for the ANOVA comparing means of the positive affect scale, *F*(2,82) = 4.17, *p* = 0.019, as well as for the ANOVA comparing means of the negative affect scale, *F*(2,82) = 24.33, *p* < 0.001; therefore, the Brown-Forsythe *F*-ratios are reported for both ANOVAs. Analyzing the positive affect scale there was a significant main effect, *F*(2,74.05) = 111.28, *p* < 0.001, η_p_ = 0.726 (for means see **Table 2**). *Post hoc* Gabriel Tests revealed that the positive film group showed significantly more positive affect than the neutral film group, *p* < 0.001, as well as significantly more positive affect than the negative film group, *p* < 0.001. The neutral film group displayed significantly more positive affect than the negative film group, *p* < 0.001. Looking at the negative affect scale there was a significant main effect, *F*(2,30.8) = 121.4, *p* < 0.001, η_p_ = 0.750 (for means see **Table 2**). *Post hoc* Gabriel Tests revealed that the negative film group showed significantly more negative affect than the neutral film group, *p* < 0.001, as well as significantly more negative affect than the positive film group, *p* < 0.001. The neutral and the positive film groups did not differ significantly from one another, *p* = 0.98.

**Table 2:** Mean positive affect (PA) and negative affect (NA) scores of the Emotion Report Form for all three experimental conditions in Experiment 2a.

	Negative film (*n* = 28)	Neutral film (*n* = 30)	Positive film (*n* = 27)
PA	1.31 ± 1.36	3.78 ± 1.51	6.45 ± 0.89
NA	3.35 ± 1.50	0.17 ± 0.37	0.09 ± 0.10


*Data are presented as means ± SD.*

###### Global–Local-Visual Processing Task

In order to examine the effects of emotions on the attentional scope, mean numbers of global choices in the Global–Local-Visual Processing Task were subjected to a one-way ANOVA with the factor FILM. Since the Levene’s Test, *F*(2,82) = 4.35, *p* = 0.016, revealed that homogeneity of variances was violated, the Brown-Forsythe *F*-ratio is reported. There was a significant main effect for the factor FILM indicating differences between the positive, *M* = 3.33, *SD* = 2.87, the neutral film, *M* = 1.67, *SD* = 2.29, and the negative film group, *M* = 1.36, *SD* = 1.83, *F*(2,70.5) = 5.52, *p* = 0.006, η_p_ = 0.12 (see **Table 3**). *Post hoc* Gabriel Tests revealed that the positive film group had significantly more global choices than the neutral film group, *p* = 0.028, as well as significantly more global choices than the negative film group, *p* = 0.008. The neutral and the negative film group did not differ significantly from one another, *p* = 0.944.

**Table 3:** Mean numbers of global choices (Global) in the Global–Local Visual Processing Task for experimental groups in Experiment 2a and mean numbers of named resource building (Building), social (Social), and resource protecting actions (Protecting) in the Twenty Statement Test (TST) for experimental groups in Experiment 2b.

	Negative film (*n* = 28)	Neutral film (*n* = 30)	Positive film (*n* = 27)
Global	1.36 ± 1.83	1.67 ± 2.29	3.33 ± 2.87
Building	2.86 ± 2.29	6.89 ± 3.08	7.86 ± 4.52
Social	1.50 ± 1.00	1.00 ± 0.86	3.28 ± 2.58
Protecting	4.04 ± 2.65	0.21 ± 0.79	0.14 ± 0.44


*Data are presented as means ± SD.*

##### Experiment 2b

As we conducted six separate ANOVAs in Experiment 2b, we employed the Bonferroni correction in order to reduce the risk of false positive results. Thus, the adjusted critical *p*-value for each ANOVA was *p* = 0.008 in order to maintain an experiment wise alpha level of α = 0.05.

###### Manipulation check

In order to test whether emotion induction worked as intended in Experiment 2b mean scores of the negative affect scale as well as mean scores of the positive affect scale were both subjected to a one-way ANOVA with the factor FILM. The Levene’s test indicated that the assumption of homogeneity of variance was violated for the ANOVA comparing means of the positive affect scale, *F*(2,82) = 5.4, *p* = 0.006 as well as for the ANOVA comparing means of the negative affect scale, *F*(2,82) = 21.4, *p* < 0.001; therefore, the Brown-Forsythe *F*-ratios are reported for both ANOVAs. Analyzing the positive affect scale there was a significant main effect, *F*(2,69.6) = 132.0, *p* < 0.001, η_p_ = 0.76 (for means see **Table 4**). *Post hoc* Gabriel Tests revealed that the positive film group showed significantly more positive affect than the neutral film group, *p* < 0.001, as well as significantly more positive affect than the negative film group, *p* < 0.001. Moreover, the neutral film group displayed significantly more positive affect than the negative film group, *p* < 0.001. Looking at the negative affect scale there was a significant main effect, *F*(2,43.4) = 160.6, *p* < 0.001, η_p_ = 0.79 (for means see **Table 4**). *Post hoc* Gabriel Tests revealed that the negative film group showed significantly more negative affect than the neutral film group, *p* < 0.001, as well as significantly more negative affect than the positive film group, *p* < 0.001. However, the neutral and the positive film group did not differ significantly from one another, *p* = 0.67.

**Table 4:** Mean positive affect (PA) and negative affect (NA) scores of the Emotion Report Form for all three experimental conditions in Experiment 2b.

	Negative film (*n* = 29)	Neutral film (*n* = 30)	Positive film (*n* = 26)
PA	1.20 ± 1.21	3.58 ± 1.56	6.53 ± 0.78
NA	3.73 ± 1.31	0.33 ± 0.70	0.10 ± 0.15


*Data are presented as means ± SD.*

###### Twenty-Statement Test

To examine whether there was a broadening of the thought-action repertoire the mean numbers of ideas participants wrote down in the Twenty-Statement Test were analyzed by means of a one-way ANOVA. There were no significant differences between the positive film, *M* = 8.10, *SD* = 4.49, the neutral film, *M* = 7.68, *SD* = 2.97, and the negative film group, *M* = 6.96, *SD* = 2.37, *F*(2,82) < 1. Concerning resource building actions there was a significant difference between the positive, *M* = 7.86, *SD* = 4.52, the neutral film, *M* = 6.89, *SD* = 3.08, and the negative film group, *M* = 2.86, *SD* = 2.29, *F*(2,82) = 54.4, *p* < 0.001, η_p_ = 0.291 (see **Table 3**). *Post hoc* Tukey Tests revealed that the negative film group named significantly less resource building actions than the positive film group, *p* < 0.001, as well as significantly less than the neutral film group, *p* < 0.001. However, the neutral and the positive film group did not differ significantly from one another, *p* = 0.539. Since the Levene’s Test showed that the assumption of homogeneity of variances was not met for the mean number of social actions, *F*(2,82) = 6.11, *p* = 0.003, and the mean numbers of resource protecting actions, *F*(2,82) = 31.13, *p* < 0.001, Brown-Forsythe *F*-ratios are reported. Regarding social actions there was a significant differences between the positive, *M* = 3.28, *SD* = 2.58, the neutral film, *M* = 1.0, *SD* = 0.86, and the negative film group, *M* = 1.5, *SD* = 1.0, *F*(2,43.3) = 14.8, *p* < 0.001, η_p_ = 0.260 (see **Table 3**). *Post hoc* Gabriel Tests revealed that the positive film group named significantly more social actions than the neutral film group, *p* < 0.001, as well as significantly more social actions than the negative film group, *p* < 0.001. The negative and neutral film group, however, did not differ significantly from one another, *p* = 0.61. Looking at resources protecting actions there was a significant difference between the positive, *M* = 0.14, *SD* = 0.44, the neutral film, *M* = 0.21, *SD* = 0.79, and the negative film group, *M* = 4.04, *SD* = 2.65, *F*(2,33.3) = 53.5, *p* < 0.001, η_p_ = 0.57 (see **Table 3**). *Post hoc* Gabriel Tests showed that the negative film group named significantly more resource protecting actions than the positive film group, *p* < 0.001, as well as more resource protecting actions than the neutral film group, *p* < 0.001. However, the neutral and the positive film group did not differ significantly from one another, *p* = 0.997.

## Discussion

The purpose of Experiments 2a and 2b was to replicate Fredrickson’s and Branigan’s (2005) findings to provide further evidence for the non-causality assumption. Thus, Experiment 2a examined effects of positive and negative affect on the visual attentional scope and Experiment 2b looked at effects of positive and negative affect on the thought-action repertoire. We found a visual attentional broadening effect of positive affect in Experiment 2a, but not a visual attentional narrowing effect of negative affect. Hence, positive emotions seem to broaden the visual attentional scope. However, the affect did neither have a broadening nor a narrowing effect on the thought-action repertoire in Experiment 2b which contradicts the Broaden-and-Build Theory concerning the respective part of the theory. This becomes even more evident when looking at the content analysis of the thought-action repertoire: participants in the positive affect condition did not name significantly more potentially resource building actions than participants in the neutral affect condition. But still, we got some promising findings which may shed some light on the adaptive function of positive emotions. Participants in the positive affect condition named significantly more social actions than those in both other conditions which leads us to the conclusion that positive emotions’ main function is to make social bonding and interactions more likely. Thus, emotions indeed seem to have a behavior guiding function according to the content analysis. Moreover, as the negative film differed significantly in valence and arousal ratings from the neutral film, we are cautious in interpreting results of negative affect in the section “Discussion” since affect and arousal are confounded in the negative film group.

## General Discussion

We conducted two experiments in order to examine two contradicting key assumptions within the framework of the Broaden-and-Build Theory (Fredrickson, 2001). In Experiment 1 we tested the causality assumption which assumes broadened visual attention to be a mediator variable between positive affect and the thought-action repertoire. We did so by comparing the number and the content of named action urges between groups with a broad, neutral, and narrow visual attentional scope. Results revealed that there were no differences between groups regarding the number and the content of named action urges. Hence, these results challenge Conway et al.’s (2013) causality assumption. In Experiment 2 we tested the non-causality assumption which assumes positive emotions to affect a number of different cognitive processes without specifying the relations and interactions among those. We did so by replicating and extending Fredrickson and Branigan’s (2005) original findings. In Experiment 2a we examined effects of positive, neutral, and negative affect on the visual attentional scope. Results indicated a visual attentional broadening effect of positive emotions. In Experiment 2b we investigated effects of positive, neutral, and negative affect on the thought-action repertoire. Results showed that there were no differences between groups regarding the number of named action urges. Furthermore, participants in the positive affect condition did not name more potentially resource building actions compared to the neutral affect condition. Thus, these results support the attentional broadening part and at the same time challenge the resource building part of the Broaden-and-Build Theory. However, the results obtained from the theory driven content analysis of the Twenty-Statement-Test point to a guiding function of positive affect, which seems to promote social action urges. Thus, these findings provide initial evidence that the ambit of the Broaden-and-Build Theory is too general and should, therefore, be specified. Finally, it is important to note that main effects of valence in our study can only be interpreted for positive affect since the negative film differed significantly in the arousal dimension from both other films.

There is additional empirical evidence for the broadening effect of positive emotions supporting our findings (Wadlinger and Isaacowitz, 2006; Johnson et al., 2010; Yegiyan and Yonelinas, 2011; Uddenberg and Shim, 2015). For example, a recent meta-analysis by Pool et al. (2016) found a general global attentional bias (Hedges’ *g* = 0.258) for positive emotions especially in tasks that included early attentional processing and stimuli of personal relevance. Furthermore, other studies go along with our results, thus, leading to the conclusion that positive emotions do seem to broaden the attentional focus. There is also empirical evidence concerning our finding that positive emotions promote social action urges, however, the empirical basis is not as strong either. There are studies which find that positive emotions predicted increased social support (Schiffrin and Falkenstern, 2012; Lin, 2015) or prosocial behavior (Andreychik and Migliaccio, 2015; Cavanaugh et al., 2015; Rand et al., 2015). Hence, keeping this relatively small empirical basis in mind, results concerning the endorsement of social action urges by positive emotions have to be interpreted with caution, too.

What are the implications of our results for the Broaden-and-Build Theory? We found positive emotions and not broadened visual attention to guide the content of the thought-action repertoire. A mere manipulation of the visual attentional scope did not lead to the same effect. However, positive affect did influence the visual attentional scope as well as the thought-action repertoire at the same time. Nevertheless, it did not do so in a sequentially mechanistic way, as Conway et al. (2013, p. 18) assume, but rather in a more complex way. Thus, our data clearly favor the non-causality assumption over the causality assumption. Still, the non-causality assumption is far away from being specific. This means future studies need to further explore how the different cognitive processes might interact with one another in order to lead to the proposed resource building outcome. However, when looking at our data we do not see a difference in the number of potentially resource building actions between the positive and the neutral film condition. Thus, it seems questionable whether positive emotions really endorse resource building actions more than neutral mental states do. Looking out for potentially resource building actions could rather be a usually active mental function that could be inhibited under particular circumstances. This goes along with the quartet theory of human emotions (Koelsch et al., 2015) which links the amygdala to this kind of activation-inhibition phenomenon. In short, normally engaging in potentially resource building actions, while inhibiting this kind of behavior, e.g., only in high arousing dangerous situations, would be far more adaptive than just engaging in resource building actions after experiencing a positive emotion. Hence, our data do not support the assumption that the evolutionary adapted function of positive emotions is the building of general personal resources, but rather point toward an increase of social action urges. In turn, this would mean that positive emotions prepare our cognitive system to be social and to connect to other people resulting in enhanced everyday prosocial behavior (e.g., Andreychik and Migliaccio, 2015). This could explain the adaptive benefit of positive emotions since prosocial behavior within-groups increases their members’ survival chances (Boyd and Richerson, 2009).

As we have pointed out before, the above described conclusions have to be treated carefully due to the relatively small empirical basis and some limitations to our experiments we will address now. First, in Experiment 2 we just had one film clip for each addressed emotional state. Hence, the observed effects could be due to particular attributes of the presented films and not positive and negative affect in general. We could show, for example, that the negative film differed significantly in the arousal dimension which limits interpretability of results produced by the negative condition. Future experiments should use different film clips and replicate our findings. Furthermore, all combinations of high and low arousal as well as positive and negative valence should be covered by the used stimuli. This would result in a more detailed picture of arousal valence interaction effects. Second, we just used the Global–Local Visual Processing Task to measure the momentary attentional scope. However, it has to be mentioned that methods for measuring the breadth of the attentional scope are reliable, but not related to each other (Dale and Arnell, 2013). Therefore, other measures of the attentional scope could have resulted in a different outcome. This could mean that the observed attentional effects could be due to methodological issues. Future experiments should also measure the visual attentional scope using other methods like Navon letters (Dale and Arnell, 2013) or EEG measures (Vanlessen et al., 2013).

In our opinion, future research should work on making the Broaden-and-Build Theory more precise regarding how the involved cognitive processes interact with one another as well as which resource components are mainly addressed by positive emotions. We solved the question whether broadened visual attention is responsible for the alternation of the thought-action repertoire. However, we still do not know how positive emotions alter the thought-action repertoire. Future experiments could address this problem by examining effects of emotions on a neurocomputational level. However, a theory from which precise hypotheses could be derived is unfortunately still missing. Another important issue that should be addressed in future research is our postulation that healthy individuals are normally engaging in resource building actions unless this function is inhibited. Solving this matter is of a central importance to the ambit of the Broaden-and-Build Theory since Fredrickson and Branigan (2005) state that positive emotions lead to the building of more personal resources than other neutral mental states do.

On the basis of the present empirical findings, we conclude that specifying the interactions of the various cognitive processes influenced by positive affective states is a fruitful avenue for future theorizing and empirical research on the Broaden-and-Build Theory.

## Author Contributions

JR and DJ developed the experiments, analyzed the data and wrote the paper. DJ collected the data.

## Conflict of Interest Statement

The authors declare that the research was conducted in the absence of any commercial or financial relationships that could be construed as a potential conflict of interest.

## Footnotes

1 http://www.neurobs.com

2 http://www.cedrus.com
